# Medical education: microeconomics or macroeconomics?

**DOI:** 10.11604/pamj.2014.18.11.4334

**Published:** 2014-05-02

**Authors:** Kieran Walsh

**Affiliations:** 1 BMJ Learning, London, UK; 2 BMJ Publishing Group, London, UK

**Keywords:** Medical education, cost, economics

## Editorial

Medical education is expensive. [1] Worldwide the spending on medical education is likely to be over 100 million dollars. [2] This expense has led to a growing interest in ensuring that whatever is spent on medical education delivers value for money to the payer. Value for money may be evaluated by means of a number of different analyses: these include but are not limited to cost effectiveness, cost benefit and cost utility studies. [3] However the question remains: who is the payer? Generally the payer will be the individual or the government or a mixture of the two. These two stakeholders will naturally have different priorities and how we look at the issue of cost and value in medical education will depend on the stakeholder involved. There is a clear parallel with the study of microeconomics and macroeconomics. Can we learn from looking at cost in medical education in macroeconomic and microeconomic terms? I would argue that we can and that the lessons learned can have significant implications for the field.

Let's look at microeconomics first. Microeconomics enables the study of the economic activities of individuals who make decisions about relatively small resources. This is most usually in a context where individuals purchase products or services. Individuals make purchasing decisions based on price, quantity and quality and so microeconomics examines these factors in detail. Price, quantity and quality are inextricably related and so microeconomics examines all three and examines the effect that each has on the others. Applying microeconomics to medical education forces the analyst to think in a particular way. Prospective medical students for example might look at the cost of university tuition, the length of the course and its quality. The prospective student might measure quality in economic terms – that is, they might prefer a course that will be likely to get them on track to a high earning specialty. In microeconomic terms prices are always relative and so even high tuition fees might seem more than manageable when the student sees a career that can help them earn those fees many times over in a short time frame. Microeconomics also looks at concepts such as asymmetric information and choice under uncertainty – both of which have implications for medical education. The prospective medical students must make choices under conditions of relative uncertainty and worse must sometimes experience being on the wrong side of asymmetric information. When this occurs the buyer and the seller have different levels of information. Often although not always the seller has more information than the buyer. In medical education a medical school might charge high tuition fees based more on its reputation that on the quality of its course. However the prospective student might not know this.

Macroeconomics by contrast is usually defined in conventional terms as the “sum total of economic activity, dealing with the issues of growth, inflation and unemployment”. [4] At a national or international level, macroeconomics deals with the performance of an economy as a whole. Macroeconomics provides models to help countries understand and influence economic growth and to develop economic policy. Once again a macroeconomic perspective can be applied to medical education. A country and its medical schools should ideally be looking at their population and population demographics in terms of healthcare. This should help it decide the number of medical graduates that it will need in the future. If its population is growing and/or ageing, it will likely need more graduates. A macroeconomic perspective should help the country provide just the right number of graduates so that there is neither a shortage of doctors on the one hand or medical unemployment on the other. The macroeconomic perspective should ultimately influence policy at a national level. The policy might play out with a result of more medical schools or more medical schools with a primary care ethos – if that is the type of healthcare professional required. Macroeconomics and macroeconomic policies are at their core concerned with outcomes and not outcomes at an individual level but rather aggregate outcomes.

So when looking at the economics of medical education, should we adopt a macroeconomic or microeconomic approach? Broadly it is likely that a macroeconomic approach would be best. Microeconomics have their place in evaluating cost and value in medical education but this place will always be limited. According to Holburt Waring in 1925, “I think I may state that without exception it is impossible to carry on satisfactorily any form of scientific instruction, such as is necessary in medicine, on fees which are paid by the student alone.” [5] The same is likely to be true today. Even in countries with the highest tuition fees, those fees only pay for part of the cost of medical education. In any case a microeconomic approach works at an individual level and individuals will most of the time act in their own best interests. This is only natural and to be expected. However this approach will ultimately result in individuals seeing their investment in medical education as a personal investment and one that they as individuals will need to see financial returns. At its worse this could result in individuals leaving their country of origin to pursue a medical career in a country that offers them greater monetary rewards. This works at an individual level but cannot work at a country level and this leads us to look closely at the purpose of medical education. Surely the purpose is clear - medical education is not an isolated academic discipline - its sole purpose is to provide doctors that the population needs. And this can only be achieved at a population level. A macroeconomic approach to medical education will result in adequate numbers of healthcare professional education programmes that the population needs with little or no medical unemployment and thus little financial or human wastage.

Macroeconomics in medical education will not solve all of our problems but should enable us to view these problems in a new light. These problems might be a shortage of healthcare professionals overall or a shortage in certain specialties or in certain territories. These issues are current in the Western world, in emerging economies and particularly so in the world's poorest countries. The problems will not be solved by thinking in microeconomic terms. Put simply we have got to “think big”.
